# Physical Activity and Hypertension

**DOI:** 10.31083/j.rcm2309302

**Published:** 2022-09-05

**Authors:** Peter Hayes, Alexandra Ferrara, Aoife Keating, Kathryn McKnight, Andrew O'Regan

**Affiliations:** ^1^School of Medicine, Health Research Institute, University of Limerick, V94 T9PX Limerick, Ireland

**Keywords:** physical activity, exercise, cardiorespiratory fitness, hypertension, blood pressure, preventative medicine, lifestyle medicine, cardiovascular disease

## Abstract

Hypertension and physical inactivity are leading causes of premature mortality. While both are modifiable risk factors for cardiovascular disease, their prevalence remains high. As populations grow older, they are more likely to develop hypertension and to become less physically active. Scientific advances have contributed to understanding of how physical activity improves blood pressure and the clinically relevant ambulatory blood pressure, but this is not reflected in hypertension guidelines for clinical management of hypertension. The aim of this paper is to clearly present up to date knowledge from scientific studies that underpin the role of physical activity in hypertension management. Longitudinal studies in this review demonstrate a protective effect of higher physical activity levels as well as higher levels of cardiorespiratory fitness. Interventional studies report improvements in blood pressure associated with aerobic, resistance and concurrent exercise; the improvements in some studies were greatest among participant groups with established hypertensions; the effect was observed for groups with treatment-resistant hypertension also, a clinically important subgroup. The most recent research provides evidence for the synergy between physical activity and pharmacotherapy for the treatment of hypertension, providing an opportunity for clinicians to promote physical activity as an adjunctive treatment for hypertension as well as a preventative strategy. This review critiques the evidence and summarises the most up to date literature in the field of physical activity and hypertension.

## 1. Introduction

### 1.1 Hypertension

Hypertension is the main modifiable risk factor for preventing cardiovascular disease (CVD) and death [1, 2]. Poorly controlled blood pressure (BP) increases the incidence of CVD, end stage kidney disease, and all cause death/CVD death [3, 4, 5]. Elevated Ambulatory Blood Pressure Monitoring (ABPM) readings [6, 7, 8], Home Blood Pressure Monitoring (HBPM) readings [9], and office based readings [10], lead to higher incidence of CVD. The finding is consistent across age and ethnic groups [11, 12]. Furthermore, Framingham data demonstrates the effects of elevated BP on increasing incidences of coronary artery disease, cerebrovascular accidents, peripheral artery disease and heart failure with a reduced ejection fraction [13]. On the other hand, small improvements in BP control, even to the levels of 2 mmHg in systolic BP, lead to reductions in CVD events [14, 15]. A person is considered hypertensive, if systolic blood pressure (SBP) is persistently greater or equal to 140 mmHg and/or diastolic blood pressure (DBP) is persistently greater or equal to 90 mmHg; and, in the USA, stage 1 hypertension is defined as a SBP of 130–139 mmHg or DBP of 80–89 mmHg [16].

The prevalence of hypertension amongst the adult population is estimated at 31–38% [17, 18], with greater than 1 billion people globally diagnosed with the condition; two thirds of those with hypertension live in low- and middle-income countries [19]. Prevalence rises with age: in the elderly, it may be as high as 64%, with only 45% aware that their blood pressure readings are elevated. Only half of those diagnosed with hypertension take the medications prescribed to treat the condition, and of those who are treated, only half have their blood pressure optimally controlled [20]. The proportion of hypertensive persons that have their BP controlled may be as low as 20% in some cohorts [19, 21].

Analysis of the National Health and Nutrition Survey (NHANES) database demonstrates significantly higher all-cause, stroke and CVD disease mortality for uncontrolled hypertensives compared to controlled hypertensives or normotensives; notably, no significant differences in mortality were observed between controlled hypertensives and normotensives [22]. Treating and preventing elevated BP is a major public health priority. To this end, lifestyle measures—addressing physical activity, diet, body mass index and alcohol—have proven benefit for BP reduction [23]. In the 1950s, Morris and colleagues reported reduced death from cardiovascular disease among active London bus conductors compared to their seated driver colleagues [24], and the evidence base for addressing physical inactivity for the prevention and treatment of chronic conditions and prevention premature mortality has accelerated in recent years.

### 1.2 Physical Activity

The Lancet refers to a pandemic of physical inactivity [25]; it is the fourth biggest cause of mortality worldwide, and 1.4 billion people are at risk of developing or exacerbating chronic illness due to being physically inactive [26]. There is strong evidence for the association between physical inactivity and mortality, risk of cancer, depression, dementia and more chronic illnesses [27]. The WHO guidelines for physical activity and sedentary behaviour reflect significant advances in scientific knowledge relating to physical activity and health [28]. The guidelines state that a minimum of 150 minutes of moderate intensity physical activity or 75 minutes vigorous intensity physical activity per week are required for health. They emphasise a reduction in sitting time and replacing this with any physical activity. Significantly, strength training on at least two days per week is also recommended for all adults. Internationally, there is a consensus on the weekly minimum ‘dose’ of physical activity: the 2018 joint European Society of Cardiology/European Society of Hypertension guidelines recommend 30 minutes of moderate intensity physical activity on five to seven days per week in addition to resistance training on two to three days per week [29].

Some of the terminology used in this field is important to clarify:

• physical activity refers to any bodily movement involving skeletal muscles and energy expenditure [30];

• exercise is a subset of physical activity that is “planned, structured, and repetitive and has as a final or an intermediate objective the improvement or maintenance of physical fitness” [30];

• cardiorespiratory fitness is another term for aerobic fitness and is most commonly measured by V02 max (the maximum volume of oxygen that can be used);

• aerobic training is a form of training that involves cardiovascular conditioning, e.g., walking, swimming and cycling;

• resistance training is a form of exercise to strengthen muscles by working them against a weight or force;

• isometric resistance training is a form of resistance training where the muscles work without changing length, e.g., a ‘wall sit’ – holding a seated position against a wall;

• dynamic resistance training is a form of resistance training where the muscles work by contracting, resulting in the movement of a body part, e.g., a squat;

• concurrent resistance training is a combination of aerobic and dynamic resistance training;

• light intensity physical activity refers to activities such as household tasks;

• moderate physical activity equates to a brisk walk;

• vigorous physical activity includes activities like running [28].

Understanding of how physical activity benefits health is advancing rapidly; research reports that even small amounts of physical activity can reduce mortality [31]. Analysis of data from studies using accelerometers to measure physical activity reports that physical activity, at any level, is associated with reduced mortality and that the relationship is non-linear dose response, with greatest benefits observed for getting the most physically inactive somewhat active [32]. Furthermore, recognition of the importance of cardiorespiratory fitness (CRF) in preventing adverse health outcomes is growing [33]. Medical organisations have called on clinicians to record physical activity [34] and CRF [33] as risk factors on patient health records—a view endorsed by the Lancet series on physical activity [35]. Researchers and clinicians grounded in exercise medicine advocate for exercise prescribing [36, 37].

While knowledge of the devastating effects of hypertension is known and accepted, and evidence for the role of physical activity in its management is steadily emerging, it appears that the prominence of physical activity is understated in guidelines and underutilised as a primary and secondary prevention strategy among clinicians. However, this year European cardiology societies have advocated more research to promote personalised exercise prescriptions for the prevention and management of hypertension [38]. The aim of this paper is to clearly present up to date knowledge from scientific reports that underpin the role of physical activity/exercise in hypertension management. Recognising the emergence of CRF as a related factor, we will include evidence for its role where relevant. For physicians, exercise is a management strategy to be used in conjunction with pharmacological therapy as opposed to a direct choice between the two; therefore, the synergy between pharmacological and physical activity approaches. The following sections will present epidemiological studies, interventional studies and underlying physiological mechanisms and will conclude with recommendations for future directions.

## 2. Epidemiology of Physical Activity and Hypertension

Hypertension is more commonly diagnosed in females, and a significant factor in this is a higher rate of healthcare utilisation [39]. Hypertension is also more common among lower socio-economic groups, particularly among people with lower educational levels [40]. On the other hand, physical activity levels are higher among males and higher socio-economic groups; race, genetics, interpersonal and environmental factors all contribute to physical activity behaviour across the lifespan [41]. Age is a recognised risk factor for the development of hypertension, and in the National Health and Nutrition Survey, over 60% of older adults reported a diagnosis of hypertension [42]. In terms of physical activity, 1.4 billion people are insufficiently active [26], and longitudinal studies from Europe [43, 44], the USA [45] and the UK [46] report a decline in physical activity with age.

A systematic review and meta-analysis of longitudinal cohort studies investigated the relationship between physical activity and incident hypertension [47]. It included 330,222 individuals and 67,698 cases of incident hypertension. For subjects who achieved minimum weekly physical activity guideline requirements, a 6% reduction (relative risk, 0.94; 95% confidence interval, 0.92–0.97) in incident hypertension was observed. The authors concluded that additional protective benefits against hypertension were accrued with higher levels of physical activity.

Huai and colleagues conducted a meta-analysis of longitudinal studies involving 136,846 persons who were normotensive at the outset, with 15,607 developing hypertension [48] during follow up. The study reported an inverse dose response relationship between recreational physical activity levels and the risk of developing hypertension. When comparing high versus low physical activity levels, the risk reduction was 0.81: RR, 0.81; (95% CI); and, for moderate versus low, risk reduction was 0.89 (95% CI). No significant relationship was noted between physical activity accumulated at work and hypertension risk. A longitudinal study involving over 5000 participants with a 20-year follow-up period reported that both cardiorespiratory fitness and physical activity levels are inversely associated with the development of hypertension [49].

A separate review of longitudinal studies examined the relationship between physical activity and CVD mortality among subjects with established hypertension [50]. It reported that inactive individuals had double the risk of mortality compared to active. However, the authors cautioned about lack of consistency in reporting of intensity, duration and type of exercise, as well as BP status. Recently, using data from the UK Biobank study on 39,294 individuals with hypertension, researchers reported an inverse dose-relationship between mortality and accelerometer-measured physical activity [51]. The study had a median follow-up time of 6.25 years, and utilised accelerometer-measured physical activity, factors that support the reliability of the findings. Further, the authors noted that higher levels of physical activity were not associated with harm, a finding that should encourage clinicians to promote physical activity to individuals with hypertension.

The evidence base is advancing also for CRF and BP; Holmlund and colleagues observed that a large improvement in CRF was associated with an 11% lower risk of incident hypertension compared to people who maintained the same fitness levels; the finding was reported after adjustment for smoking, BMI, diet, stress and exercise habits [52]. This study involved a retrospective analysis conducted at two time points on the Swedish Health Profile Assessment database, involving 91,728 participants with a mean age of 40.7 years. Similarly, a contemporaneous systematic review and meta-analysis reported an inverse dose-response relationship between CRF and risk of developing hypertension [53]. In that review, the high CRF group had a 37% risk reduction compared to the low CRF group; and, the moderate CRF group had a 15% risk reduction compared to the low CRF group. Interestingly, BMI was noted as a modifier in the relationship, suggesting that those with higher BMI may gain more from becoming more cardiorespiratory fit. The review involved nine large cohort studies but could not distinguish between effects for gender. Furthermore, the authors acknowledged heterogeneity of how CRF measurement between studies [53]. Similarly, a longitudinal study of adolescents reported an inverse relationship between CRF and SBP [54]. The implication for the clinician is that encouraging patients to become physically fit across the lifespan is likely to prevent, or at least delay, the onset of hypertension and its negative health sequelae.

## 3. Interventional Studies

### 3.1 Exercise Modalities

#### 3.1.1 Aerobic Exercise

**Fig. 1.**
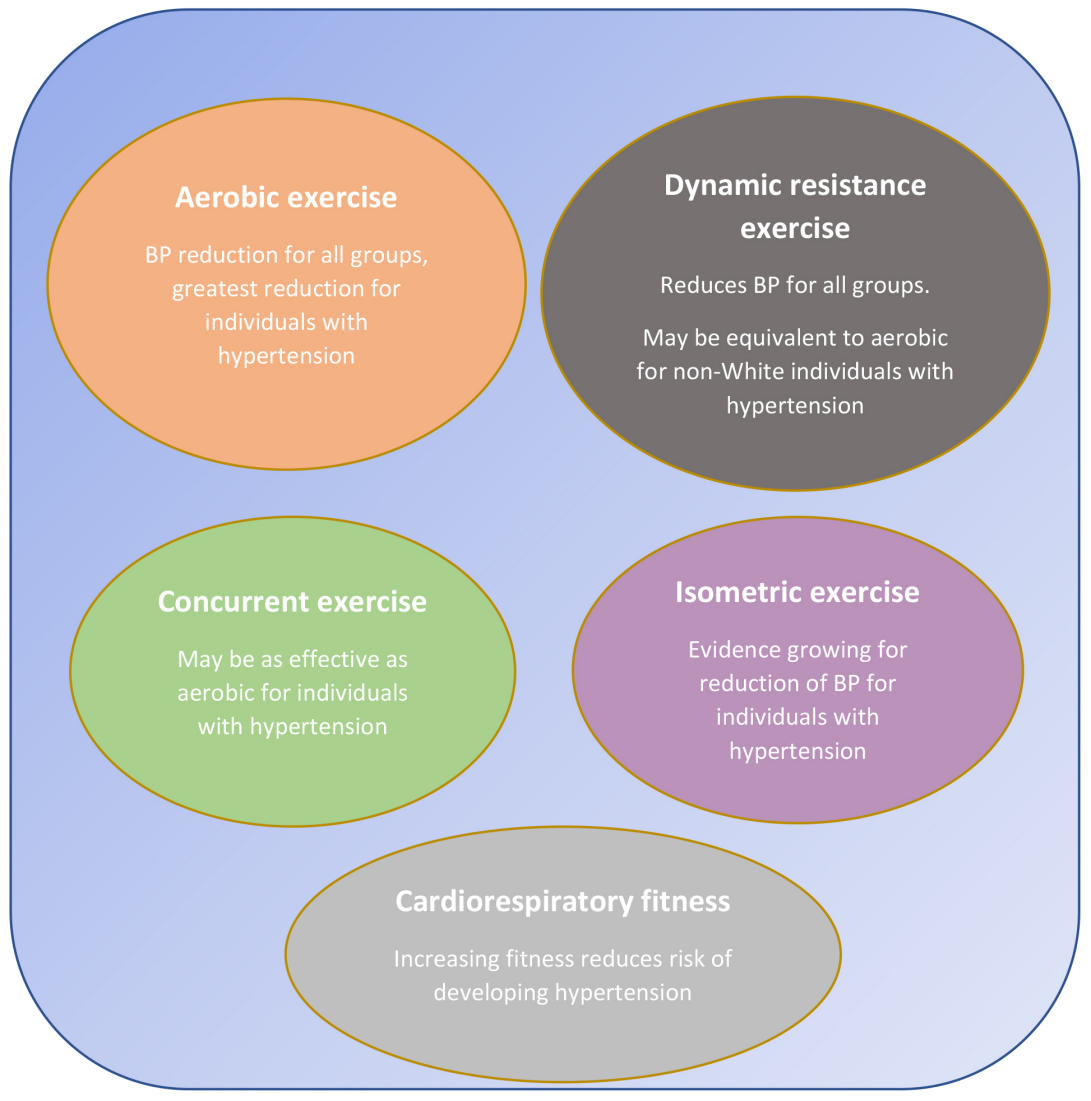
**Physical activity options for BP reduction**.

For millennia, aerobic exercise, particularly walking, has been recommended for cardiovascular prevention and longevity. Up until the past decade most research concerning physical activity and blood pressure focused on aerobic exercise. Accordingly, aerobic exercise has been recommended by systematic reviews and guidelines as the most appropriate form of physical activity for BP reduction [55]. Further large reviews of exercise, including aerobic exercise are discussed in the following sections. Some of the larger studies published recently are presented in Table 1 (Ref. [56, 57, 58, 59, 60, 61, 62, 63, 64, 65, 66, 67, 68, 69, 70]); Fig. 1 outlines key findings from the studies.

**Table 1.** **Recent trials for exercise modalities and blood pressure reducation**.

Study, year	Intervention	Participant profile, number	Outcome
Aerobic continuous v inactive control			
Williamson [64], 2022	Aerobic (moderate-high continuous)	Young normotensive adults (N = 203)	No differences in ABPM between control and aerobic exercise cohorts at 16 weeks or 52 weeks
	3 × 60-minute sessions; inactive control		
	× 16 weeks		
Blumenthal [62], 2021	Aerobic (moderate continuous)	Treatment resistant hypertensives (N = 140)	ABPM SBP was reduced in the aerobic cohort [–7.0 (95% CI, –8.5 to –4.0) mmHg], no change in control (*p* = 0.001)
	As part of cardiac rehabilitation and in conjunction with dietary input		
	Control		
	× 4 months		
Lopes [63], 2021	Aerobic (moderate continuous)	Treatment resistant hypertensives (N = 53)	ABMP SBP for the aerobic group was reduced by 7.1 mmHg (95% CI, –12.8 to –1.4; *p* = 0.02). Additionally, 24-hour ambulatory diastolic ABPM DBP was reduced (–5.1 mmHg; 95% CI, –7.9 to –2.3; *p* = 0.001)
	3 × 40-minute sessions/week		
	× 12 weeks		
Dimeo [61], 2012	Aerobic (moderate continuous)	Treatment resistant hypertensives (N = 50)	SBP reduced in the aerobic cohort [6 mmHg (+/–12 mmHg) *p* = 0.03] and DBP reduced in the aerobic cohort [3 mmHg (+/–7 mmHg) *p *< 0.03]
	Control		
	× 8–10 weeks		
Aerobic continuous v isometric interventions			
Goessler [65], 2018	Aerobic (moderate continuous) 150-minutes of CME/week	Young normotensive adults (N = 61)	Similar improvements in office SBP and ABPM
	Isometric daily 2-minute contractions (× 2 repetitions) × 8 weeks		Office DBP improved only in aerobic cohort
Pagonas [66], 2017	Aerobic (moderate continuous) 3–5 × 30-minutes sessions × 12 weeks	Hypertensive adults (N = 75)	No statistical improvement was noted in the isometric cohort
	Isometric 5 × 2-minute contractions (× 2 repetitions) × 12 weeks		A significant SBP reduction was noted in the aerobic cohort (*p* = 0.025) using ABPM
HIIT interventions			
Reljic [67], 2020	HIIT 2 sessions (cycling)	Mixed hypertensive and non-hypertensive (N = 65)	Reduced SBP [–12 mmHg (95% CI –16 to –8 mmHg), *p *< 0.001] and DBP [–10 mmHg (95% CI –13 to –7 mmHg), *p *< 0.001] were noted in the HIIT cohort
	Inactive control cohort		
	× 8 weeks		Control cohort was unchanged
Ghardashi Afousi [68], 2018	HIIT 3 sessions (cycling) v Aerobic (moderate continuous) 42-minutes × 3/week	Hypertensive and pre hypertensive adults (N = 75)	Both groups significantly improved SBP compared to control: HIIT [–3.66 mmHg] and CMIT [–3.7 mmHg]
	Inactive control		Both groups significantly improved DBP compared to control: HIIT [–5.5 mmHg] and CMIT [–4.8 mmHg] (*p *< 0.005)
	× 12 weeks		
Shepherd [69], 2015	HIIT 3 sessions	Mixed hypertensive and non-hypertensive (N = 90)	Reduced SBP [–4 mmHg (–7 to –2 mmHg), *p* = 0.01] was noted in the Moderate continuous group only
	(cycling)/week		
	Continuous aerobic		No changes were noted in the HIIT cohort; no changes were noted for DBP
	5 × 3—45-minute sessions/week		
	× 10 weeks		
Isometric exercise interventions v inactive control			
Correia [56], 2020	Isometric 3 × 2-minutes (× 4 repetitions) sessions	Mainly hypertensive (N = 102)	Reduced DBP [–3 mmHg, *p* = 0.04] in the isometric cohort
	× 8-weeks		Reduced SBP in isometric cohort but not statistically significant
Farah [57], 2018	Isometric 3 × 2-minutes (× 4 repetitions)	Medicated hypertensive adults (N = 72)	Reduced SBP from [132 mmHg +/–4 mmHg to 120 mmHg +/3 mmHg] and reduced DBP [71 mmHg+/–2 mmHg to 66+/–2 mmHg (*p *< 0.05)]
	Supervised v home-setting		
	Inactive control		
	× 12 weeks		
Medication v non – medication trial			
Mora-Rodriguez [70], 2022	3 HIIT sessions/week	Hypertensive adults (N = 178)	The medicated and non-medicated group each demonstrated similar reductions in BP:
	× 16 weeks	Medicated (n = 103)	Medicated group [–15 mmHg (95% CI –12 to –19); *p *< 0.001]; No medication cohort [–13 mmHg (95% CI –9 to –16); *p *< 0.001]
		No medication (n = 75)	The higher the baseline SBP the greater the reduction after training
Combined exercise			
Ruangthai [60], 2019	Aerobic (moderate) × 3 60-minute session/week	Mixed hypertensive and normotensive (N = 54)	SBP reduced significantly in the aerobic (–7.9%, *p *< 0.05) and combined (8.2%, *p *< 0.01) at end of intervention
	Strength × 3 dynamic resistance sessions/week		At 12-week follow-up post intervention, only the combined cohort maintained significant SBP reduction (–7.5%, *p *< 0.01)
	Combined (aspects of both within a single session)		
	× 12 weeks		
Lima [58], 2017	Aerobic group × 3 20–30 minute-sessions/week	Normotensive (N = 44)	SBP (wake and sleep) decreased similarly for the aerobic cohort and combined cohort, measured by ABPM (*p *< 0.01)
	Combined group – as above plus dynamic resistance exercise circuit		DBP (wake only) decreased by 4 mmHg in both the aerobic and combined cohorts
	True control		No change was seen for night DBP [58]
	× 10-weeks		
Sousa [59], 2013	Aerobic group (moderate-vigorous) × 3/week	Mixed hypertensive and normotensive (N = 59)	SBP reduced in the aerobic cohort [149.4 mmHg (±25 mmHg) to 134.6 mmHg (±20.1 mmHg)] and was reduced to a greater degree in the combined cohort [148.5 mmHg (±15.1 mmHg) to 124.5 mmHg (±16.5 mmHg) (*p *< 0.01)]
	Combined group – as above with one session replaced with dynamic resistance exercise × 3/week		
	Control group		DBP reduced in the aerobic cohort [80.4 mmHg (±7.6 mmHg) to 74.6mmHg (±7.9 mmHg)] and DBP reduced in the combined cohort [82.8 mmHg (±9.6 mmHg) to 71.3 mmHg (±8.0 mmHg) (*p *< 0.05)]
	× 9 months		

#### 3.1.2 Aerobic Exercise: High Intensity Interval Training (HIIT) versus Moderate Intensity Continuous Training (MICT)

The question of whether HIIT or MICT training is most effective for blood pressure has been the subject of four systematic reviews. Leal and colleagues investigated differences in BP reduction between HIIT and MICT, reviewing 15 trials of individuals with hypertension (mean age 55.8 years): HIIT and MICT reduced SBP with comparable effect but HIIT was more effective at reducing DBP for this population [71]. Another review involving 14 trials that were mainly composed of younger and middle aged adults reported a greater effect for HIIT in the hours following the bout of exercise, but no difference on nocturnal BP [72]. A systematic review conducted on studies involving an elderly population, concluded that both modalities were effective with comparative success [73].

#### 3.1.3 Resistance Training

Cornelissen and colleagues (2011) examined 28 randomized-controlled trials (with 33 study groups and 1012 participants) in a systematic review and meta-analysis [74]. Only five of the 33 study groups had hypertension—all others were either normotensive or pre-hypertensive. Dynamic resistance training (squats, push ups, bicep curls) was the dominant intervention in trials (n = 25 trials), and static resistance training (isometric hand grip) was examined in three trials. The authors remarked that the quality of trial reporting was poor overall (all were published before 2010). The combined effect for all modalities of resistance training were SBP (–3.9, 95% CI, –6.2; –1.5)/DBP (–3.6, 95% CI, –5.0; –2.1) mmHg, (*p *< 0.001). Interestingly, these results were not statistically significant in the five groups that were hypertensive at baseline. However, an important nuance arises here: some of those who were deemed pre-hypertensive pre 2010 would now be called hypertensive under current AHA guidelines. Interestingly, isometric hand grip resistance training resulted in a statistically significant larger decrease in blood pressure (–13.5 SBP/–6.1 DBP mmHg) than dynamic resistance training (–2.8 SBP/–2.7 DBP mmHg).

Cornelissen and Smart repeated the meta-analysis in 2013 to include more recent studies and widened the inclusion criteria to include dynamic aerobic endurance (jogging, swimming), dynamic and isometric resistance, and combined resistance-endurance training [75]. Overall, it involved 93 trials (105 endurance, 29 dynamic resistance, 14 combined, and 5 isometric resistance groups), with 5223 participants (3401 exercise and 1822 control). SBP was reduced after endurance (–3.5 mmHg; 95% CI –4.6 to –2.3), dynamic resistance (–1.8 mmHg; 95% CI –3.7 to –0.011), and isometric resistance (–10.9 mmHg; 95% CI –14.5 to –7.4) but not after combined training. Reductions in DBP were seen in all four modalities examined. Once again, isometric resistance training was the most effective modality reported but, interestingly, BP reductions after endurance training were greater (*p *< 0.0001) in 26 study groups of hypertensive persons (–8.3 [–10.7 to –6.0]/–5.2 [–6.8 to –3.4] mmHg) than in 50 groups of pre-hypertensive persons (–2.1 [–3.3 to –0.83]/–1.7 [–2.7 to –0.68]) and 29 groups of persons with normotension (–0.75 [–2.2 to +0.69]/–1.1 [–2.2 to –0.068]). The key point from Cornelissen and Smart’s review is that aerobic exercise has the strongest research base, with 105 endurance groups included, and aerobic exercise will reduce BP among normotensive, high normal and hypertensive groups; greatest BP reductions are among individuals with hypertension.

MacDonald’s systematic review of dynamic resistance training included 64 studies, reported a modest SBP and DBP reduction, and concluded that the greatest reduction was observed among non-White hypertensives [76]. A recent systematic review, focussing specifically on isometric exercise and blood pressure, concluded that this exercise modality has a statistically significant and clinically important SBP lowering effect [77]. Two large trials investigated hand grip training (a form of isometric exercise) for individuals with hypertension and prehypertension; compared to inactive controls, statistically significant reductions in DBP [56, 57] were reported in both, and SBP reported in one [57].

#### 3.1.4 Concurrent Exercise Training

A meta-analysis of 68 trials reported that concurrent resistance training is as useful as aerobic exercise for the for reducing BP in people with hypertension [78]. Utilising ABPM for measurement, Lima and colleagues compared aerobic, combined and inactive control; they reported similar SBP reductions for both during wake, as well as similar sleep and similar waking DBP reductions [58]. A second trial, conducted among an older cohort with more hypertension at baseline, reported a greater SBP and DBP reduction among the combined exercise cohort [59]. Most interestingly, the third study reported similar SBP reductions for aerobic and combined cohorts (but no significant reduction for strength only); the SBP reduction was maintained at 12-weeks follow-up. In practical terms, this is relevant as the unsupervised period during which benefits were maintained is reflective of real-life settings, where in most instances continuous support is not available [60].

### 3.2 Exercise Dose

#### 3.2.1 Frequency 

There is no agreed frequency of ‘exercise dose’ for achieving BP reduction. Lee & Chae reported that optimum frequency of physical activity for BP lowering effect is three times per week [79]. For SBP, effect sizes depending on exercise frequency were: –6.10 mmHg (95% CI = –8.83 to –3.37) for twice weekly; –9.16 mmHg (95% CI = –11.66 to –6.66) for three times weekly’ and –6.96 mmHg (95% CI = –9.27 to –4.64) for four or more times weekly. For DBP, effect sizes based on exercise frequency were: –4.38 mmHg (95% CI = –7.09 to –1.66) for twice weekly; –5.55 mmHg (95% CI = –6.87 to –4.23) for three times weekly; and, –4.50 mmHg (95% CI = –6.80 to –2.20) for four times weekly. Cornelissen & Smart suggest that even less than three exercise sessions per week provides maximum BP benefit; this meta-analysis included various frequencies of exercise intensity, ranging from 1 to 7 times per week across 93 trials (n = 5223) [75]. In the trials reviewed for this paper, most aerobic interventions were 3–5 times per week, whereas HIIT sessions were 2–3 times weekly.

#### 3.2.2 Intensity

Lee & Chae categorized aerobic training intensity into low-intensity, moderate-intensity, and vigorous intensity in their meta-analysis [79]. They reported that only moderate- [–8.97 mmHg (95% CI = –11.11 to –6.84)] and vigorous- [–6.85 mmHg (95% CI = –12.03 to –1.66)] intensity exercise improved SBP. A similar trend was observed regarding DBP: [–5.75 mmHg (95% CI = –7.18 to –4.32)] for moderate-intensity exercise; and, for vigorous-intensity exercise [–4.36 mmHg (95% CI = –5.94 to –2.79)]. Moderate-intensity exercise had the most impact on reduction of both SBP and DBP. This is consistent with findings from Cornelissen & Smart, who reported lower intensity training to be associated with the smallest effect size on SBP (*p* = 0.032) and DBP (*p* = 0.03) [75]. Their meta-analysis involved trials with intensity from 35% to 95% peak oxygen consumption for endurance training, between 30% and 100% of 1-repetition maximum for dynamic resistance training, and between 10% and 40% for isometric resistance training.

#### 3.2.3 Duration

Lee & Chae report that intervention duration is important in mediating BP reductions of aerobic physical activity [79]. This meta-analysis included trials with intervention duration varying between 4 and 37 weeks. Their results show that exercise durations of 4–7 weeks [–3.04 mmHg (95% CI = –5.14 to –0.95)], 8–11 weeks [–9.12 mmHg (95% CI = –14.09 to –4.16)], 12–23 weeks [–8.77 mmHg (95% CI = –11.06 to –6.49)] and 24 or more weeks [–8.24 mmHg (95% CI = –10.77 to –6.27)] all reduce SBP and DBP (–3.5 mmHg: 4–7 weeks, –5.4 mmHg; 8–11 weeks, –4.8 mmHg: 12–23 weeks, –7 mmHg: 24 or more weeks). The intervention duration of 8–11 weeks showed the greatest effect in reducing SBP whereas 24 weeks or longer had the most impact in lowering DBP. These findings contrast to the results of Cornelissen & Smart (2013) who found program duration of <24 weeks appears to lower DBP (*p *< 0.01) and SBP (*p *< 0.0001) to a greater extent than programs of >24 weeks’ duration [75]. Cao *et al*. [80] suggested that shorter programs <8 weeks produced the most profound reductions in SBP [–16.66 mmHg (95% CI: –18.55 to –14.76), *p *< 0.05], whereas DBP was optimised at durations of exercise >24 weeks [–7.52 mmHg (95% CI: –12.42 to –2.62), *p *< 0.05].

### 3.3 Ambulatory Blood Pressure (ABP) Measurement

In 2020, a review conducted by Saco-Ledo and colleagues reported that 24-hour blood pressure, DBP and night-time blood pressure [81]. Two interesting and, perhaps unexpected, findings were noted from subgroup analyses: firstly, significant improvements in ABP were only noted for participants treated with anti-hypertensive medication and secondly only aerobic exercise produced a significant improvement [81]. A separate review involving 17 study groups and 633 participants reported ABP improvements with exercise for daytime but not night-time BP [82].

### 3.4 Baseline Blood Pressure Categories 

A meta-analysis conducted by Faggard and Cornelissen, that included 72 RCTs and 105 study groups, investigated the effects of exercise training on BP. The authors concluded that the greatest reductions in BP were observed among hypertensive study groups [83]. The finding was consistent with a later systematic review and meta-analysis by Cornelissen and Smart, that grouped a total of 5223 participants into hypertensive (26 study groups), pre-hypertensive (50 study groups) and normotensive (29 study groups) [75]. The study reported statistically higher SBP and DBP reductions among the hypertensive group, followed by the pre-hypertensive group. However, current guidelines have reclassified what was pre-hypertension as hypertension, making interpretation of older reports more complex [84]. A systematic review reported greater benefits from exercise for people with pre-hypertension compared to normotensives, but that all categories demonstrated improvements [85].

A recent review focussed only on older hypertensive adults, involving 69 RCTs and 2272 participants [86]; it reported significant decreases in both SBP and DBP after exercise. Similarly, a contemporaneous review of training in normotensive adults demonstrated that exercise produced beneficial effects on BP [87].

#### Treatment Resistant Hypertension

Treatment resistant hypertension is associated with increased organ damage and was defined as having uncontrolled blood pressure despite taking three anti-hypertensive medications or having controlled blood pressure and taking at least four anti-hypertensives; it has been the subject of high-quality trials and systematic reviews. An RCT used ABPM to measure the impact of an 8–10-week programme on treatment resistant hypertension: significant reductions in SBP and DBP were observed [61]. Recently, a larger trial involving 140 individuals with treatment resistant hypertension reported significant improvements in SBP as measured by ABPM [62]. In 2021, two trials with a combined total of 193 individuals with treatment resistant hypertension, were randomised to a moderate intensity aerobic exercise intervention or a control; the trials were of three to four months in duration and both reported statistically significant ABPM measured SBP reductions close to 7 mmHg [62, 63]. A systematic review, published in 2022, involving 10 studies and a combined population of 380 participants, observed that exercise appears to be effective in reducing BP for people with treatment resistant hypertension [88].

### 3.5 Synergy Between Exercise and Pharmacotherapy

It is likely that synergy will exist if both these treatment modalities are optimized in single patients. In 2020, Magalhães reported that aerobic exercise significantly increased urinary levels of ACE (angiotensin converting enzyme) and plasma levels of ACE2. This may indicate the synergistic mechanism of renin-angiotensin-aldosterone inhibitor based anti-hypertensive drugs and exercise [89]. Whilst many trials based on hypertensive patients have shown that aerobic exercise reduced BP [82, 90], most have not attempted to peel this back and assess effects of medication, aerobic exercise or a combination of both.

A network meta-analysis investigating the efficacy of exercise versus medications in lowering BP suggested that medications are more effective (mean difference –3.96 mmHg, 95% CI –5.02 to –2.91); the authors noted that all types of exercise lowered BP and that individuals who were taking anti-hypertensive medications had the best BP lowering responses [91]. A second network meta-analysis on this topic acknowledged that ‘the current evidence base with a bias towards medication research may partly explain the circumspection around the efficacy of exercise in guidelines and practice’ [92].

While a recent systematic review purported that the benefits of exercise and pharmacotherapy are not additive and are not synergistic [93], a series of small trials have demonstrated improvements in BP from combining exercise with pharmacological management. The most recent of these (which post-dated the review) concluded that both medication and physical activity (in this instance HIIT) have independent and additive effects in lowering BP (24 hour Mean Arterial Pressure: –5.7 mmHg) [94].

Current evidence suggests that pharmacologically treated hypertensive people should aim to exercise. This may reduce the need for escalation of anti-hypertensive drug doses or the prescription of additional drugs over time, as BP remains controlled. Issues remain on the generalisability of knowledge from these trials to real world settings-as in these trials most patients were well controlled hypertensive patients, largely taking renin-angiotensin-aldosterone inhibitor drugs, and participant numbers remain small. Further large trials are required and should include hypertensive populations that are sub-optimally controlled (between 130/80 to 145/90 mmHg etc.), and those with co-morbidities.

## 4. Mechanisms Mediating the Relationship between Physical Activity and Blood Pressure

### 4.1 Postexercise Hypotension

Postexercise hypotension (PEH), the phenomenon whereby a single session of exercise can generate a transient reduction in BP [95], which occurs in both hypertensive and normotensive individuals [96], but seems to have a greater and longer lasting effect in the latter group [97]. The primary mechanism proposed in PEH is the central baroreflex pathway, and this is mediated by a complex system involving an exercise-induced neuroplasticity in the brainstem medulla [98]. During exercise, the sympathetic system is activated, initiating catecholamine release, inducing peripheral vasoconstriction [99], thereby raising BP. Subsequent cessation of exercise initiates reactivation of parasympathetic mechanisms and sympathetic deactivation [100]. Importantly from a clinical perspective, sustained elevations in BP (as in hypertensives), have been found to alter the central baroreflex pathway by resetting to higher activation threshold [101], thereby reducing reflex sensitivity.

### 4.2 Sustained Vasodilation 

Sustained postexercise vasodilation refers to the persistence of increased blood flow to vascular beds following exercise cessation; local vasodilatory mechanisms - namely nitric oxide (NO) release and histamine activation pathway are implicated in sustained postexercise vasodilation [102].

NO is produced and released by endothelial cells, exhibiting vasodilatory effects through vascular smooth muscle relaxation and sympathetic neural constrictor antagonism [103]. Exercise stimulates NO release through increased shear stress leading to endothelial cell deformation [104, 105]. The hypotensive effect mediated by NO release in response to exercise appears to be related to exercise intensity [106] and may have genetic and gender influences. Positive effects on BP, mediated by increases in NO levels have been reported for normotensive [107] as well as hypertensive [108] populations.

Activation of skeletal muscles, as occurs during exercise, promotes the formation and release of histamine, a chemical that plays a key role in sustained postexercise vasodilation [109, 110]. Research also points to two other potential mediators of physical activity on BP: angiogenesis, whereby physical activity is responsible for morphological changes in the vascular tree, thereby augmenting blood flow; and the sensitising effect of physical activity on insulin [111]. A contemporaneous review reported that aerobic exercise improves vascular remodelling across the layers of the arterial wall, hence reducing peripheral vascular resistance, by reducing inflammation, proliferation and fibrosis [112].

## 5. Discussion

Physical inactivity and hypertension are extremely common, and both are drivers for increased cardiovascular morbidity and mortality. Engagement in any modality of physical activity has a BP lowering effect, but a combination of moderate to vigorous intensity aerobic, in conjunction with resistance training, over at least three days of the week, appears to be the most successful. The greatest BP lowering effects are seen in those who are already deemed to have high BP. Hypertension is more common in the elderly, and in those from lower socio-economic groups. Getting these specific groups more active will ultimately benefit the individual and society. Incorporating behaviour change into healthcare consultations is key to doing this. The promotion of the prescription of exercise as a treatment modality is ever-increasing, but further work is needed-it is not suggested however the physical activity alone should be a substitute for the use of anti-hypertensive medication. Nor do we suggest that healthcare professionals should be expected to shoulder the enormous task of increasing population physical activity levels alone – physical inactivity is a complex, multi-level public health problem that requires a strategic multi-system response, including co-operation between state departments such as transport, urban planning and education, as well as regional and local organisations.

A problem identified in this review is the heterogeneity of studies. While several high-quality systematic reviews and meta-analyses of both RCTs and longitudinal studies exist, major disparities exist between population groups. Physiology, genetics, gender, age and morbidity levels are likely correlates in the relationship between physical activity/exercise and hypertension. However, with most trials involving healthy people with normal BP, the effect reported will be lower than it would be if a homogenous population with hypertension was recruited. Further research should involve high quality, well powered, RCTs examining those with grade 1 hypertension (<160 mmHg SBP, <100 mmHg DBP) and those with multimorbidity. The authors recommend the use of ABP monitoring and accelerometery to objectively measure variables.

It is also clear further examination of the synergistic effect of medication plus increased exercise needs analysis as a clear scientific basis for this hypothesis now exists. Although at an early stage of investigation-it is likely that some synergy exists between anti-hypertensive medication and increased exercise in reducing BP. From a clinical viewpoint, physicians routinely promote exercise as well as prescribing medications for hypertension.

## 6. Future Directions

Technology is broadening the scope of research in this field; home BP monitoring is an effective way of reducing BP and could effectively be managed with the collaboration of healthcare professionals; exploring how this can be done safely and effectively, perhaps by involving synchronous and asynchronous mechanisms, is a priority for clinical researchers. Medical organisations are advocating for investment in technology as well as training and reimbursement for healthcare professionals to build health information capacity. In this context, wearables have become commonplace and have potential to bring about awareness of personal physical inactivity and to motivate and bring about behavioural change in relation to physical activity and exercise. Technology can facilitate data collection on BP and physical activity levels, and healthcare professionals of the future will need to be adaptable in order to harness its potential. Through judicious use of technology, future research could provide evidence to inform more precise recommendations for the physical activity needed for BP reductions. With the rapid development of devices and opportunities, an exciting future awaits clinicians and researchers in the fields of physical activity and hypertension.

## 7. Conclusions

Robust evidence from RCTs and systematic reviews supports exercise for the management and treatment of hypertension. All types of physical activity produce blood pressure lowering effects and improvements are observed for people in every blood pressure category. Traditionally, evidence was strongest for aerobic exercise, but as research in this field expands, resistance (both dynamic and isometric) and concurrent exercise, have demonstrated improvements in blood pressure. Achieving healthy blood pressure levels is a major health challenge; to achieve this, clinicians have sufficient evidence to encourage patients to become more physically active, as part of a wider strategy.

## References

[1] Lawes CM, Vander Hoorn S, Rodgers A (2008). Global burden of blood-pressure-related disease, 2001. *Lancet*.

[2] Ezzati M, Vander Hoorn S, Lawes CM, Leach R, James WP, Lopez AD (2005). Rethinking the “diseases of affluence” paradigm: global patterns of nutritional risks in relation to economic development. *PLoS Medicine*.

[3] Lewington S, Clarke R, Qizilbash N, Peto R, Collins R (2002). Age-specific relevance of usual blood pressure to vascular mortality: a meta-analysis of individual data for one million adults in 61 prospective studies. *Lancet*.

[4] Britton KA, Gaziano JM, Djoussé L (2009). Normal systolic blood pressure and risk of heart failure in us male physicians. *European Journal of Heart Failure*.

[5] Kalaitzidis RG, Bakris GL (2010). Prehypertension: is it relevant for nephrologists. *Kidney International*.

[6] Staessen JA (1999). Predicting Cardiovascular Risk Using Conventional vs Ambulatory Blood Pressure in Older Patients with Systolic Hypertension. *Journal of the American Medical Association*.

[7] Dolan E, Stanton A, Thijs L, Hinedi K, Atkins N, McClory S (2005). Superiority of Ambulatory over Clinic Blood Pressure Measurement in Predicting Mortality. *Hypertension*.

[8] Myers MG (2011). Implications of Ambulatory Blood Pressure Monitoring Substudies on the Interpretation of Clinical Trials in Hypertension: should the Threshold for Drug Therapy be Lower in Older Patients. *The Journal of Clinical Hypertension*.

[9] Shimbo D, Abdalla M, Falzon L, Townsend RR, Muntner P (2016). Studies comparing ambulatory blood pressure and home blood pressure on cardiovascular disease and mortality outcomes: a systematic review. *Journal of the American Society of Hypertension*.

[10] Ettehad D, Emdin CA, Kiran A, Anderson SG, Callender T, Emberson J (2016). Blood pressure lowering for prevention of cardiovascular disease and death: a systematic review and meta-analysis. *The Lancet*.

[11] Lawes CM, Rodgers A, Bennett DA, Parag V, Suh I, Ueshima H (2003). Blood pressure and cardiovascular disease in the Asia Pacific region. *Journal of Hypertension*.

[12] Brown D, Giles W, Greenlund K (2007). Blood Pressure Parameters and Risk of Fatal Stroke, NHANES II Mortality Study. *American Journal of Hypertension*.

[13] Peeters A (2002). A cardiovascular life history. *European Heart Journal*.

[14] Stamler J (1993). Blood Pressure, Systolic and Diastolic, and Cardiovascular Risks. *Archives of Internal Medicine*.

[15] Lim SS, Vos T, Flaxman AD, Danaei G, Shibuya K, Adair-Rohani H (2012). A comparative risk assessment of burden of disease and injury attributable to 67 risk factors and risk factor clusters in 21 regions, 1990–2010: a systematic analysis for the Global Burden of Disease Study 2010. *The Lancet*.

[16] Bakris G, Ali W, Parati G (2019). ACC/AHA Versus ESC/ESH on Hypertension Guidelines. *Journal of the American College of Cardiology*.

[17] Mills KT, Bundy JD, Kelly TN, Reed JE, Kearney PM, Reynolds K (2016). Global Disparities of Hypertension Prevalence and Control. *Circulation*.

[18] Danon-Hersch N, Marques-Vidal P, Bovet P, Chiolero A, Paccaud F, Pécoud A (2009). Prevalence, awareness, treatment and control of high blood pressure in a Swiss city general population: the CoLaus study. *European Journal of Cardiovascular Prevention & Rehabilitation*.

[19] members N-R (2021). Worldwide trends in hypertension prevalence and progress in treatment and control from 1990 to 2019: a pooled analysis of 1201 population-representative studies with 104 million participants. *Lancet*.

[20] Murphy CM, Kearney PM, Shelley EB, Fahey T, Dooley C, Kenny RA (2016). Hypertension prevalence, awareness, treatment and control in the over 50s in Ireland: evidence from the Irish Longitudinal Study on Ageing. *Journal of Public Health*.

[21] Wolf-Maier K, Cooper RS, Kramer H, Banegas JR, Giampaoli S, Joffres MR (2004). Hypertension Treatment and Control in Five European Countries, Canada, and the United States. *Hypertension*.

[22] Zhou D, Xi B, Zhao M, Wang L, Veeranki SP (2018). Uncontrolled hypertension increases risk of all-cause and cardiovascular disease mortality in us adults: the NHANES III Linked Mortality Study. *Scientific Reports*.

[23] Williams B, Mancia G, Spiering W, Agabiti Rosei E, Azizi M, Burnier M (2018). 2018 Practice guidelines for the management of arterial hypertension of the European Society of Cardiology and the European Society of Hypertension. *Blood Pressure*.

[24] Morris JN, Heady JA, Raffle PAB, Roberts CG, Parks JW (1953). Coronary heart-disease and physical activity of work. *The Lancet*.

[25] Kohl HW, Craig CL, Lambert EV, Inoue S, Alkandari JR, Leetongin G (2012). The pandemic of physical inactivity: global action for public health. *The Lancet*.

[26] Guthold R, Stevens GA, Riley LM, Bull FC (2018). Worldwide trends in insufficient physical activity from 2001 to 2016: a pooled analysis of 358 population-based surveys with 1·9 million participants. *The Lancet Global Health*.

[27] Cunningham C, O’ Sullivan R, Caserotti P, Tully MA (2020). Consequences of physical inactivity in older adults: a systematic review of reviews and meta‐analyses. *Scandinavian Journal of Medicine & Science in Sports*.

[28] World Health Organisation (2020). WHO guidelines on physical activity and sedentary behaviour. https://www.who.int/publications/i/item/9789240015128.

[29] Williams B, Mancia G, Spiering W, Agabiti Rosei E, Azizi M, Burnier M (2018). 2018 ESC/ESH Guidelines for the management of arterial hypertension: The Task Force for the management of arterial hypertension of the European Society of Cardiology (ESC) and the European Society of Hypertension (ESH). *European Heart Journal*.

[30] Caspersen CJ, Powell KE, Christenson GM (1985). Physical activity, exercise, and physical fitness: definitions and distinctions for health-related research. *Public Health Reports*.

[31] Hupin D, Roche F, Gremeaux V, Chatard J, Oriol M, Gaspoz J (2015). Even a low-dose of moderate-to-vigorous physical activity reduces mortality by 22% in adults aged ≥60 years: a systematic review and meta-analysis. *British Journal of Sports Medicine*.

[32] Ekelund U, Tarp J, Steene-Johannessen J, Hansen BH, Jefferis B, Fagerland MW (2019). Dose-response associations between accelerometry measured physical activity and sedentary time and all cause mortality: systematic review and harmonised meta-analysis. *British Medical Journal*.

[33] Kaminsky LA, Arena R, Beckie TM, Brubaker PH, Church TS, Forman DE (2013). The Importance of Cardiorespiratory Fitness in the United States: the need for a National Registry. *Circulation*.

[34] Thompson WR, Sallis R, Joy E, Jaworski CA, Stuhr RM, Trilk JL (2020). Exercise is Medicine. *American Journal of Lifestyle Medicine*.

[35] Reis RS, Salvo D, Ogilvie D, Lambert EV, Goenka S, Brownson RC (2016). Scaling up physical activity interventions worldwide: stepping up to larger and smarter approaches to get people moving. *The Lancet*.

[36] Sallis RE (2009). Exercise is medicine and physicians need to prescribe it. *British Journal of Sports Medicine*.

[37] O’Regan A, Pollock M, D’Sa S, Niranjan V (2021). ABC of prescribing exercise as medicine: a narrative review of the experiences of general practitioners and patients. *BMJ Open Sport & Exercise Medicine*.

[38] Hanssen H, Boardman H, Deiseroth A, Moholdt T, Simonenko M, Kränkel N (2022). Personalized exercise prescription in the prevention and treatment of arterial hypertension: a Consensus Document from the European Association of Preventive Cardiology (EAPC) and the ESC Council on Hypertension. *European Journal of Preventive Cardiology*.

[39] Zhang Y, Moran AE (2017). Trends in the Prevalence, Awareness, Treatment, and Control of Hypertension among Young Adults in the United States, 1999 to 2014. *Hypertension*.

[40] Leng B, Jin Y, Li G, Chen L, Jin N (2015). Socioeconomic status and hypertension. *Journal of Hypertension*.

[41] Bauman AE, Reis RS, Sallis JF, Wells JC, Loos RJ, Martin BW (2012). Correlates of physical activity: why are some people physically active and others not. *The Lancet*.

[42] Ong KL, Cheung BMY, Man YB, Lau CP, Lam KSL (2007). Prevalence, Awareness, Treatment, and Control of Hypertension among United States Adults 1999–2004. *Hypertension*.

[43] Murtagh EM, Murphy MH, Murphy NM, Woods C, Nevill AM, Lane A (2015). Prevalence and correlates of physical inactivity in community-dwelling older adults in Ireland. *PLoS ONE*.

[44] Bennie JA, Chau JY, van der Ploeg HP, Stamatakis E, Do A, Bauman A (2013). The prevalence and correlates of sitting in European adults - a comparison of 32 Eurobarometer-participating countries. *International Journal of Behavioral Nutrition and Physical Activity*.

[45] Schrack JA, Zipunnikov V, Goldsmith J, Bai J, Simonsick EM, Crainiceanu C (2014). Assessing the “physical cliff”: detailed quantification of age-related differences in daily patterns of physical activity. *Journals of Gerontology Series A: Biomedical Sciences and Medical Sciences*.

[46] Lindsay Smith G, Banting L, Eime R, O’Sullivan G, van Uffelen JGZ (2017). The association between social support and physical activity in older adults: a systematic review. *International Journal of Behavioral Nutrition and Physical Activity*.

[47] Liu X, Zhang D, Liu Y, Sun X, Han C, Wang B (2017). Dose–Response Association between Physical Activity and Incident Hypertension. *Hypertension*.

[48] Huai P, Xun H, Reilly KH, Wang Y, Ma W, Xi B (2013). Physical Activity and Risk of Hypertension. *Hypertension*.

[49] Carnethon MR, Evans NS, Church TS, Lewis CE, Schreiner PJ, Jacobs DR (2010). Joint Associations of Physical Activity and Aerobic Fitness on the Development of Incident Hypertension. *Hypertension*.

[50] Rossi A, Dikareva A, Bacon SL, Daskalopoulou SS (2012). The impact of physical activity on mortality in patients with high blood pressure. *Journal of Hypertension*.

[51] del Pozo Cruz B, Ahmadi M, Inan‐Eroglu E, Huang B, Stamatakis E (2022). Prospective Associations of Accelerometer‐Assessed Physical Activity with Mortality and Incidence of Cardiovascular Disease among Adults with Hypertension: the UK Biobank Study. *Journal of the American Heart Association*.

[52] Holmlund T, Ekblom B, Börjesson M, Andersson G, Wallin P, Ekblom-Bak E (2021). Association between change in cardiorespiratory fitness and incident hypertension in Swedish adults. *European Journal of Preventive Cardiology*.

[53] Cheng C, Zhang D, Chen S, Duan G (2021). The association of cardiorespiratory fitness and the risk of hypertension: a systematic review and dose–response meta-analysis. *Journal of Human Hypertension*.

[54] Agostinis-Sobrinho C, Ruiz JR, Moreira C, Abreu S, Lopes L, Oliveira-Santos J (2018). Cardiorespiratory Fitness and Blood Pressure: a Longitudinal Analysis. *The Journal of Pediatrics*.

[55] Pescatello LS, MacDonald HV, Ash GI, Lamberti LM, Farquhar WB, Arena R (2015). Assessing the Existing Professional Exercise Recommendations for Hypertension: a Review and Recommendations for Future Research Priorities. *Mayo Clinic Proceedings*.

[56] A. Correia M, Oliveira PL, Farah BQ, Vianna LC, Wolosker N, Puech‐Leao P (2020). Effects of Isometric Handgrip Training in Patients with Peripheral Artery Disease: a Randomized Controlled Trial. *Journal of the American Heart Association*.

[57] Farah BQ, Rodrigues SL, Silva GO, Pedrosa RP, Correia MA, Barros MV (2018). Supervised, but not home-based, isometric training improves brachial and central blood pressure in medicated hypertensive patients: a randomized controlled trial. *Frontiers in Physiology*.

[58] Lima LG, Bonardi JTM, Campos GO, Bertani RF, Scher LML, Moriguti JC (2017). Combined aerobic and resistance training: are there additional benefits for older hypertensive adults. *Clinics*.

[59] Sousa N, Mendes R, Abrantes C, Sampaio J, Oliveira J (2013). A randomized 9-month study of blood pressure and body fat responses to aerobic training versus combined aerobic and resistance training in older men. *Experimental Gerontology*.

[60] Ruangthai R, Phoemsapthawee J (2019). Combined exercise training improves blood pressure and antioxidant capacity in elderly individuals with hypertension. *Journal of Exercise Science & Fitness*.

[61] Dimeo F, Pagonas N, Seibert F, Arndt R, Zidek W, Westhoff TH (2012). Aerobic Exercise Reduces Blood Pressure in Resistant Hypertension. *Hypertension*.

[62] Blumenthal JA, Hinderliter AL, Smith PJ, Mabe S, Watkins LL, Craighead L (2021). Effects of Lifestyle Modification on Patients with Resistant Hypertension: Results of the TRIUMPH Randomized Clinical Trial. *Circulation*.

[63] Lopes S, Mesquita-Bastos J, Garcia C, Bertoquini S, Ribau V, Teixeira M (2021). Effect of Exercise Training on Ambulatory Blood Pressure among Patients with Resistant Hypertension. *JAMA Cardiology*.

[64] Williamson W, Lewandowski AJ, Huckstep OJ, Lapidaire W, Ooms A, Tan C (2022). Effect of moderate to high intensity aerobic exercise on blood pressure in young adults: the TEPHRA open, two-arm, parallel superiority randomized clinical trial. *EClinicalMedicine*.

[65] Goessler KF, Buys R, VanderTrappen D, Vanhumbeeck L, Cornelissen VA (2018). A randomized controlled trial comparing home-based isometric handgrip exercise versus endurance training for blood pressure management. *Journal of the American Society of Hypertension*.

[66] Pagonas N, Vlatsas S, Bauer F, Seibert FS, Zidek W, Babel N (2017). Aerobic versus isometric handgrip exercise in hypertension. *Journal of Hypertension*.

[67] Reljic D, Frenk F, Herrmann HJ, Neurath MF, Zopf Y (2020). Low-volume high-intensity interval training improves cardiometabolic health, work ability and well-being in severely obese individuals: a randomized-controlled trial sub-study. *Journal of Translational Medicine*.

[68] Ghardashi Afousi A, Izadi MR, Rakhshan K, Mafi F, Biglari S, Gandomkar Bagheri H (2018). Improved brachial artery shear patterns and increased flow-mediated dilatation after low-volume high-intensity interval training in type 2 diabetes. *Experimental Physiology*.

[69] Shepherd SO, Wilson OJ, Taylor AS, Thøgersen-Ntoumani C, Adlan AM, Wagenmakers AJ (2015). Low-volume high-intensity interval training in a gym setting improves cardio-metabolic and psychological health. *PLoS ONE*.

[70] Mora-Rodriguez R, Ortega JF, Morales-Palomo F, Ramirez-Jimenez M, Moreno-Cabañas A, Alvarez-Jimenez L (2022). Endurance exercise training reduces blood pressure according to the Wilder’s principle. *International Journal of Sports Medicine*.

[71] Leal JM, Galliano LM, Del Vecchio FB (2020). Effectiveness of High-Intensity Interval Training Versus Moderate-Intensity Continuous Training in Hypertensive Patients: a Systematic Review and Meta-Analysis. *Current Hypertension Reports*.

[72] Marcal IR, Goessler KF, Buys R, Casonatto J, Ciolac EG, Cornelissen VA (2021). Post-exercise hypotension following a single bout of high intensity interval exercise vs. a single bout of moderate intensity continuous exercise in adults with or without hypertension: a systematic review and meta-analysis of randomized clinical trials. *Frontiers in Physiology*.

[73] Carpes L, Costa R, Schaarschmidt B, Reichert T, Ferrari R (2022). High-intensity interval training reduces blood pressure in older adults: a systematic review and meta-analysis. *Experimental Gerontology*.

[74] Cornelissen VA, Fagard RH, Coeckelberghs E, Vanhees L (2011). Impact of Resistance Training on Blood Pressure and other Cardiovascular Risk Factors. *Hypertension*.

[75] Cornelissen VA, Smart NA (2013). Exercise Training for Blood Pressure: a Systematic Review and Meta‐analysis. *Journal of the American Heart Association*.

[76] MacDonald HV, Johnson BT, Huedo‐Medina TB, Livingston J, Forsyth KC, Kraemer WJ (2016). Dynamic Resistance Training as Stand‐alone Antihypertensive Lifestyle Therapy: a Meta‐Analysis. *Journal of the American Heart Association*.

[77] Kelley GA, Kelley KS, Stauffer BL (2021). Isometric exercise and inter-individual response differences on resting systolic and diastolic blood pressure in adults: a meta-analysis of randomized controlled trials. *Blood Pressure*.

[78] Corso LML, Macdonald HV, Johnson BT, Farinatti P, Livingston J, Zaleski AL (2016). Is Concurrent Training Efficacious Antihypertensive Therapy? A Meta-analysis. *Medicine & Science in Sports & Exercise*.

[79] Lee SH, Chae YR (2020). Characteristics of Aerobic Exercise as Determinants of Blood Pressure Control in Hypertensive Patients: a Systematic Review and Meta-Analysis. *Journal of Korean Academy of Nursing*.

[80] Cao L, Li X, Yan P, Wang X, Li M, Li R (2019). The effectiveness of aerobic exercise for hypertensive population: a systematic review and meta‐analysis. *The Journal of Clinical Hypertension*.

[81] Saco‐Ledo G, Valenzuela PL, Ruiz‐Hurtado G, Ruilope LM, Lucia A (2020). Exercise Reduces Ambulatory Blood Pressure in Patients with Hypertension: a Systematic Review and Meta‐Analysis of Randomized Controlled Trials. *Journal of the American Heart Association*.

[82] Cornelissen VA, Buys R, Smart NA (2013). Endurance exercise beneficially affects ambulatory blood pressure. *Journal of Hypertension*.

[83] Fagard RH, Cornelissen VA (2007). Effect of exercise on blood pressure control in hypertensive patients. *European Journal of Cardiovascular Prevention & Rehabilitation*.

[84] Carey RM, Whelton PK (2018). Prevention, Detection, Evaluation, and Management of High Blood Pressure in Adults: Synopsis of the 2017 American College of Cardiology/American Heart Association Hypertension Guideline. *Annals of Internal Medicine*.

[85] Pescatello LS, Buchner DM, Jakicic JM, Powell KE, Kraus WE, Bloodgood B (2019). Physical Activity to Prevent and Treat Hypertension: a Systematic Review. *Medicine & Science in Sports & Exercise*.

[86] Kazeminia M, Daneshkhah A, Jalali R, Vaisi-Raygani A, Salari N, Mohammadi M (2020). The Effect of Exercise on the Older Adult’s Blood Pressure Suffering Hypertension: Systematic Review and Meta-Analysis on Clinical Trial Studies. *International Journal of Hypertension*.

[87] Loaiza-Betancur AF, Pérez Bedoya E, Montoya Dávila J, Chulvi-Medrano I (2020). Effect of Isometric Resistance Training on Blood Pressure Values in a Group of Normotensive Participants: a Systematic Review and Meta-analysis. *Sports Health: a Multidisciplinary Approach*.

[88] Saco-Ledo G, Valenzuela PL, Ruilope LM, Lucia A (2022). Physical Exercise in Resistant Hypertension: A Systematic Review and Meta-Analysis of Randomized Controlled Trials. *Frontiers in Cardiovascular Medicine*.

[89] Magalhães DM, Nunes-Silva A, Rocha GC, Vaz LN, de Faria MHS, Vieira ELM (2020). Two protocols of aerobic exercise modulate the counter-regulatory axis of the renin-angiotensin system. *Heliyon*.

[90] de Barcelos GT, Heberle I, Coneglian JC, Vieira BA, Delevatti RS, Gerage AM (2022). Effects of Aerobic Training Progression on Blood Pressure in Individuals With Hypertension: A Systematic Review With Meta-Analysis and Meta-Regression. *Frontiers in Sports and Active Living*.

[91] Naci H, Salcher-Konrad M, Dias S, Blum MR, Sahoo SA, Nunan D (2019). How does exercise treatment compare with antihypertensive medications? a network meta-analysis of 391 randomised controlled trials assessing exercise and medication effects on systolic blood pressure. *British Journal of Sports Medicine*.

[92] Noone C, Leahy J, Morrissey EC, Newell J, Newell M, Dwyer CP (2020). Comparative efficacy of exercise and anti-hypertensive pharmacological interventions in reducing blood pressure in people with hypertension: a network meta-analysis. *European Journal of Preventive Cardiology*.

[93] Pescatello LS, Wu Y, Gao S, Livingston J, Sheppard BB, Chen M (2021). Do the combined blood pressure effects of exercise and antihypertensive medications add up to the sum of their parts? a systematic meta-review. *BMJ Open Sport & Exercise Medicine*.

[94] Ramirez‐Jimenez M, Morales‐Palomo F, Moreno‐Cabañas A, Alvarez‐Jimenez L, Ortega JF, Mora‐Rodriguez R (2021). Effects of antihypertensive medication and high‐intensity interval training in hypertensive metabolic syndrome individuals. *Scandinavian Journal of Medicine & Science in Sports*.

[95] MacDonald JR (2002). Potential causes, mechanisms, and implications of post exercise hypotension. *Journal of Human Hypertension*.

[96] Kenney MJ, Seals DR (1993). Postexercise hypotension. Key features, mechanisms, and clinical significance. *Hypertension*.

[97] Halliwill JR (2001). Mechanisms and Clinical Implications of Post-exercise Hypotension in Humans. *Exercise and Sport Sciences Reviews*.

[98] Chen C, Bonham AC (2010). Postexercise Hypotension. *Exercise and Sport Sciences Reviews*.

[99] Lyssand JS, DeFino MC, Tang XB, Hertz AL, Feller DB, Wacker JL (2008). Blood pressure is regulated by an alpha1D-adrenergic receptor/dystrophin signalosome. *Journal of Biological Chemistry*.

[100] Rosenwinkel ET, Bloomfield DM, Allison Arwady M, Goldsmith RL (2001). Exercise and autonomic function in health and cardiovascular disease. *Cardiology Clinics*.

[101] Malpas SC (2004). What sets the long-term level of sympathetic nerve activity: is there a role for arterial baroreceptors. *American Journal of Physiology-Regulatory, Integrative and Comparative Physiology*.

[102] Halliwill JR, Buck TM, Lacewell AN, Romero SA (2013). Postexercise hypotension and sustained postexercise vasodilatation: what happens after we exercise. *Experimental Physiology*.

[103] Gamboa A, Okamoto LE, Diedrich A, Choi L, Robertson D, Farley G (2012). Sympathetic activation and nitric oxide function in early hypertension. *American Journal of Physiology-Heart and Circulatory Physiology*.

[104] Nosarev AV, Smagliy LV, Anfinogenova Y, Popov SV, Kapilevich LV (2014). Exercise and NO production: relevance and implications in the cardiopulmonary system. *Frontiers in Cell and Developmental Biology*.

[105] Tinken TM, Thijssen DHJ, Hopkins N, Dawson EA, Cable NT, Green DJ (2010). Shear Stress Mediates Endothelial Adaptations to Exercise Training in Humans. *Hypertension*.

[106] Santana HAP, Moreira SR, Asano RY, Sales MM, Córdova C, Campbell CSG (2013). Exercise intensity modulates nitric oxide and blood pressure responses in hypertensive older women. *Aging Clinical and Experimental Research*.

[107] Goto C, Nishioka K, Umemura T, Jitsuiki D, Sakagutchi A, Kawamura M (2007). Acute Moderate-Intensity Exercise Induces Vasodilation through an Increase in Nitric Oxide Bioavailiability in Humans. *American Journal of Hypertension*.

[108] Nyberg M, Jensen LG, Thaning P, Hellsten Y, Mortensen SP (2012). Role of nitric oxide and prostanoids in the regulation of leg blood flow and blood pressure in humans with essential hypertension: effect of high-intensity aerobic training. *The Journal of Physiology*.

[109] Harries MG, Burge PS, O’brien I, Cromwell O, Pepys J (1979). Blood histamine levels after exercise testing. *Clinical Allergy*.

[110] Romero SA, McCord JL, Ely MR, Sieck DC, Buck TM, Luttrell MJ (2017). Mast cell degranulation and de novo histamine formation contribute to sustained postexercise vasodilation in humans. *Journal of Applied Physiology*.

[111] Gambardella J, Morelli MB, Wang X, Santulli G (2020). Pathophysiological mechanisms underlying the beneficial effects of physical activity in hypertension. *the Journal of Clinical Hypertension*.

[112] Song Y, Jia H, Hua Y, Wu C, Li S, Li K (2022). The Molecular Mechanism of Aerobic Exercise Improving Vascular Remodeling in Hypertension. *Frontiers in Physiology*.

